# Prevalence and correlates of elder neglect in the community-dwelling Chinese population: New evidence from the CLHLS study

**DOI:** 10.3389/fpubh.2023.1123835

**Published:** 2023-03-13

**Authors:** Yi-cheng Fu, Min-yue Pei, Jiao-jiao Liao, Nan Li, Fu-chun Zhang, Hui-lin Liu

**Affiliations:** ^1^Department of Geriatrics, Peking University Third Hospital, Beijing, China; ^2^Research Center of Clinical Epidemiology, Peking University Third Hospital, Beijing, China

**Keywords:** neglect, elder abuse, elder people, community, population-based study

## Abstract

**Background:**

This study aimed to explore the prevalence of elder neglect (EN) and its associated factors among community-based Chinese older adults.

**Methods:**

We used data from the 2018 phase of a nationwide cross-sectional study, the Chinese Longitudinal Healthy Longevity Survey (CLHLS), which recruited 15,854 older adults to complete the study interviews that incorporated six dimensions of EN, namely, life neglect, social isolation, medical neglect, poor living situation, family neglect, and social neglect. Multivariate logistic regression was used to explore factors associated with EN.

**Results:**

We included demographic factors, chronic diseases, cognitive function, and daily activity function in our comprehensive analysis and showed that they had different effects on the six EN dimensions. Different demographic factors such as gender, age, marriage, education, occupation, residence, and household income were included in the comprehensive analysis, and the results showed that these factors had different effects on the six dimensions of EN. Next, we found that older adults with chronic diseases are prone to life neglect, medical neglect, and residential environment neglect. Older adults with better cognitive abilities were less likely to be neglected, and a decline in daily activity capacity has been linked to EN in older adults.

**Conclusion:**

Future studies are needed to identify the health effects of these associated factors, develop prevention strategies for EN, and improve the quality of life of older adults living in communities.

## 1. Introduction

The global population is aging rapidly. A recent survey suggested that the global prevalence of elder abuse (EA) has dramatically increased from 10 to 15.7% in the 21st century (1). A growing body of research points toward the severe impact of EA on mortality, incidence rates of physical and mental complications, decreased quality of life, and social resource consumption (2). The World Health Organization (WHO) report on violence and health presented an internationally accepted definition of EA, in which EA is characterized as a single or repeated act or lack of appropriate action, occurring within any relationship where there is an expectation of trust that causes harm or distress to an older person. This concept is now widely accepted and encompasses five categories of abuse: physical, psychological or emotional, financial or material, sexual, and neglect (3).

Elder neglect (EN) is a relatively common form of EA. The global statistics on EN are still lacking due to the concealment of abuse events and screening difficulties in the phenomenon itself. The U.S. National Center on Elder Abuse (NCEA) defined EN as “the refusal or failure to fulfill any part of a person's obligations or duties to an elder.” (4). However, in China, EN is usually considered an undesirable consequence associated with impaired mental and physical wellbeing in older adults, accompanied by social dysfunction (5). There is still no clear and unifying concept of EN; the definition may differ because of socioeconomic or medical factors in different regions and countries. We refer to the conceptual framework for the etiology of self-neglect presented in previous studies and combined it with the contents of the CHLHS questionnaire to screen for EN-related factors for inclusion in our analysis (6, 7). In our study analysis, we divided elder neglect into three domains, namely, self-neglect, family neglect, and social neglect. Self-neglect includes four dimensions, namely, life neglect, social isolation, medical neglect, and poor living situation. Together with family neglect and social neglect, there are six dimensions, from which we separately analyze the data to achieve a more comprehensive and in-depth investigation of the problem of EN.

Elder neglect remains an unsolved problem in many countries. EN rates vary from country to country and region to region due to the influence of social, economic, and cultural differences. Despite the differences, EN remains the second most common type of elder abuse. In a survey of older adults in the global community (28 studies, 39 515 participants), the rate of reported EN was 4.2% (1). The United States reported a neglect rate of 5.1% (8). In Europe, reported rates of neglect ranged from 0.2 to 31.1% (9). In China, one study focused on rural areas and found that the pooled EN prevalence estimate was up to 26% (95% CI 17–35) (10). Another data from a cross-sectional study of 7,446 Chinese people, from 2009 to 2010, showed that nearly 8.73% of respondents reported that their older parents had been neglected within the last year (11). Another cross-sectional study from 2005, which included 412 urban-dwelling older adults in Nanjing city, showed that caregiver neglect is the most common form of EA, and the proportion of EN is estimated at 16.9% (12). However, epidemiological data on the Chinese population, with a large sample size and a complete follow-up over an extended period of time, are still lacking (13).

Elder neglect is accompanied by a variety of chronic diseases, such as cerebrovascular disease, cardiovascular disease, lung disease, diabetes, and cancer (14). It has been suggested that gradually accumulating psychological distress and a higher burden of medical comorbidities may exacerbate physical and cognitive dysfunction in older adults, leading to increased vulnerability to EN. These risk factors, combined with a lack of family or social support networks, magnify insufficiency in self-protective capabilities and finally result in EN (15). The primary outcomes of EN include the following three aspects: abnormal living status, impaired interpersonal relationships, and poor health outcomes. Self-neglect generally manifests itself as an older person who cannot perform daily living tasks, has poor personal hygiene and sanitation, and refuses to receive medical and social assistance (6). Poor living conditions and hygiene-related health problems are frequently encountered. Older adults who are unable to achieve adequate dietary intake or consume fresh food lose weight and become malnourished. Lack of friends, isolation, social disconnection, and loneliness are frequently reported in neglected older adults (16). When family caregivers (spouse, children, or relatives) do not have the strength or skills to meet the older person's daily needs or are unwilling to fulfill caregiving responsibilities, EN occurs (17). At the community level, home care, public health, and medical services play an important role in caring for older adults with functional impairment and self-care disabilities. Older adults who are not provided with medicines or seek timely medical treatment will experience an exacerbation of chronic conditions, which can lead to more complications, hospitalizations, or even death (18). Given this complexity, the most promising intervention to prevent EN is the construction of an inter-professional team. Practice-based evidence shows that a multidisciplinary team, consisting of community representatives, doctors, social workers, lawyers, police, and other volunteers, can be of great help in coordinating effective responses and protecting older adults against EN (19).

As previously clarified, EN in community-dwelling older adults is insidious, complicated, and difficult to detect. Despite recent advances in our knowledge, the factors influencing EN in older Chinese adults remain incompletely understood. In this article, we focus on elder neglect (EN) and discuss how new questions included within the 2018 phase of the Chinese Longitudinal Healthy Longevity Survey (CLHLS) can be used to identify community-dwelling older populations at risk of EN. Our goal was to examine the association between specific environmental and sociodemographic covariates and the affecting factors of EN in community-dwelling older adults. This is performed with the aim of helping community health services become more aware of the potential damage of EN and play a positive role in the management of social assistance to vulnerable older adults.

## 2. Methods

### 2.1. Study population and data source

This national cross-sectional study used the CLHLS cohort database. The CLHLS is a national survey and study on older adults in China. It started in 1998 and collected information on adults over 65 years of age from 22 provinces in China. The CLHLS cohort collected information about the basic status of older adults (demographic characteristics, living conditions, and household income); personality and emotional characteristics (depression and anxiety); general abilities (responsiveness, attention, and memory); lifestyle (exercise and eating habits); daily activity ability; and individual and family structure. All information was obtained through in-home interviews, lasting approximately 2 h and conducted by trained investigators; each participant provided a written informed consent form, which was signed by the next of kin if the participant was unable to write. More details about the CLHLS study design can be found elsewhere (20). This study included the 2018 data from the CLHLS cohort and analyzed the neglect of 15,854 older adults. Neglect status included six dimensions: life neglect, social isolation, medical neglect, poor living situation, family neglect, and social neglect.

### 2.2. Assessment of neglect status

Life neglect included unwillingness to cook, avoidance of fresh fruits and vegetables, irregular exercise, and poor hygiene. If one of the aforementioned four items is observed, one point is obtained, and the sum of the scores represents the score of life neglect. The scores of other neglect indicators were similar to those of life neglect, which means that the sum of the items was used to obtain the total score. Social isolation included a lack of social interaction with others, unwillingness to participate in social activities, unwillingness to share information, lack of cooperation, and solving daily problems on their own. Medical neglect included untimely access to emergency care, unaccompanied clinic visits, unattended care for illnesses, and irregular physical examinations. Poor living conditions included poor kitchen ventilation, a musty odor in the house, a leaky roof, and an untidy home environment. Family neglect included the inability to get along with spouse, living alone due to lack of assistance from children, being unattended and living in nursing homes, reluctant caregivers, and unmet ADL needs. Social neglect included relatives' inability to resolve daily problems, including the provision of social care, arranging doctors' house calls and medicine delivery, shopping help, legal aid, and handling family disputes. All the details are available in the attachment of methods in Supplementary material.

### 2.3. Assessment of related factors

Factors related to neglect were included in the study. The related factors included age, sex, marital status, years of education, residence, occupation before retirement, household income, Activities of Daily Living (ADL) score, Instrumental Activity of Daily Living (IADL) score, Minimum Mental State Examination (MMSE) score, and self-reported chronic disease. Demographic variables were grouped by reference to the CLHLS questionnaire or the classifications used in previous CLHLS studies. Participants were divided into three groups: 60–74 years; 75–84 years; and 85 years and older. Marital status was divided into two groups: spouses and widowed or divorced individuals. Education was divided into four groups, according to the number of years of education: 0 years; 1–6 years; 7–12 years; and 13 years and above. Residences were divided into two groups: city, town, or country. Occupation before retirement was divided into two groups: manual and non-manual labor. Household income was divided into three categories: ¥0–8,000; ¥8,000–30,000; and more than ¥30,000. ADL, IADL, MMSE, and self-reported chronic diseases were evaluated as the related factors. At the time of analysis, these variables were adjusted in the multivariable model.

### 2.4. Data analysis

Baseline characteristics of the study population in different groups were described as percentages for categorical variables and medians (interquartile range [IQR]) for continuous variables. We tested the statistical differences using the chi-square test for categorical variables and the non-parametric test for continuous variables (did not conform to the normal distribution). We divided the study population into two groups according to the median and used multivariate logistic regression models to analyze affecting factors related to neglect, we used all the covariates to set up the logistic regression model. Those who were higher than the median score was considered as high risk group in each dimension of EN. Odds ratios (ORs) with 95% confidence intervals (CIs) were calculated. All analyses were performed using SPSS version 26.0. All *P*-values were two-sided, with values of <0.05 revealing statistical significance.

## 3. Results

### 3.1. Sample characteristics

Table 1 presents the characteristics of the entire study population. In this study, we included 15,854 participants in the statistical analysis, and the mean age (standard deviation) of the population was 85.4 (11.7), of which more than half were older than 85 years. A total of 8942 (56.4%) were older women and less likely to live with a spouse than the male participants. Approximately 70% of the participants performed manual work before they retired, and approximately 60% of the participants were illiterate. As for the IADL score, the median (25th and 75th percentile) of the whole population was 3.00 (0.00 and 7.00), and women tended to face more difficulties in daily activities. The majority lived in a town or country, and almost half of the population had more than ¥30,000 household income per year.

**Table 1:** Baseline characteristics of CLHLS 2018 participants.

**Characteristic**	**Overall**	**Male**	**Female**	***p*-value**
	**(*****N*** = **15,854)**	**(*****N*** = **6,912)**	**(*****N*** = **8,942)**	
**Age**	85.48 (11.67)	83.33 (10.91)	87.14 (11.97)	<0.001
60–74	3,364 (21.2%)	1,742 (25.2%)	1,622 (18.1%)	
75–84	4,274 (27.0%)	2,062 (29.8%)	2,212 (24.7%)	
More than 85	8,216 (51.8%)	3,108 (45.0%)	5,108 (57.1%)	
**Marital status**				
Married/partnered	6,401 (41.0%)	3,980 (58.5%)	2,421 (27.1%)	<0.001
Widow/separated/other	9,453 (59.6%)	2,932 (42.4%)	6,521 (72.9%)	
Residence				
City	3,541 (22.3%)	1,621 (23.5%)	1,920 (21.4%)	0.003
Town or Country	12,313 (77.7%)	5,291 (76.5%)	7,022 (78.5%)	
**Education year**				
0	9,900 (62.4%)	3,179 (46.0%)	6,721 (75.2%)	<0.001
1–6	4,215 (26.6%)	2,513 (36.4%)	1,702 (19.0%)	
7–12	1,267 (8.0%)	886 (12.8%)	381 (4.3%)	
13+	472 (3.0%)	334 (4.8%)	138 (1.5%)	
**Occupation before retirement**				
Manual Labor	11,459 (72.3%)	4,478 (64.8%)	6,981 (78.1%)	<0.001
Non-Manual Labor	4,395 (27.7%)	2,434 (35.2%)	1,961 (21.9%)	
**Household income (**¥**/year)**				
0–8,000	4,560 (28.8%)	1,899 (27.5%)	2,661 (29.8%)	<0.001
8,000–30,000	3,943 (24.9%)	1,651 (23.9%)	2,292 (25.6%)	
30,000 or more	7,351 (46.4%)	3,362 (48.6%)	3,989 (44.6%)	
Hypertension	6,259 (39.5%)	2,638 (38.2%)	3,621 (40.5%)	0.003
Diabetes	1,422 (9.0%)	596 (8.6%)	826 (9.2%)	0.189
Heart disease	2,534 (16.0%)	1,012 (14.6%)	1,522 (17.0%)	<0.001
Cerebrovascular disease	1,654 (10.4%)	802 (11.6%)	852 (9.5%)	<0.001
Lung disease	1,562 (9.9%)	861 (12.5%)	701 (7.8%)	<0.001
Cancer	205 (1.3%)	107 (1.5%)	98 (1.1%)	0.015
MMSE Score	20.87 (10.73)	22.82 (10.11)	19.37 (10.96)	<0.001
IADL	3.00 (0.00, 7.00)	1.00 (0.00, 6.00)	4.00 (1.00, 8.00)	<0.001
ADL	3.00 (2.00, 3.00)	3.00 (2.00, 3.00)	3.00 (2.00, 3.00)	<0.001

Data are n (%), M (P25, P75) and mean (SD).

CLHLS, China Longitudinal Healthy Longevity Survey; ADL, activities of daily living scale; ADL, instrumental activities of daily living scale.

### 3.2. Distribution

**Figure 1:**
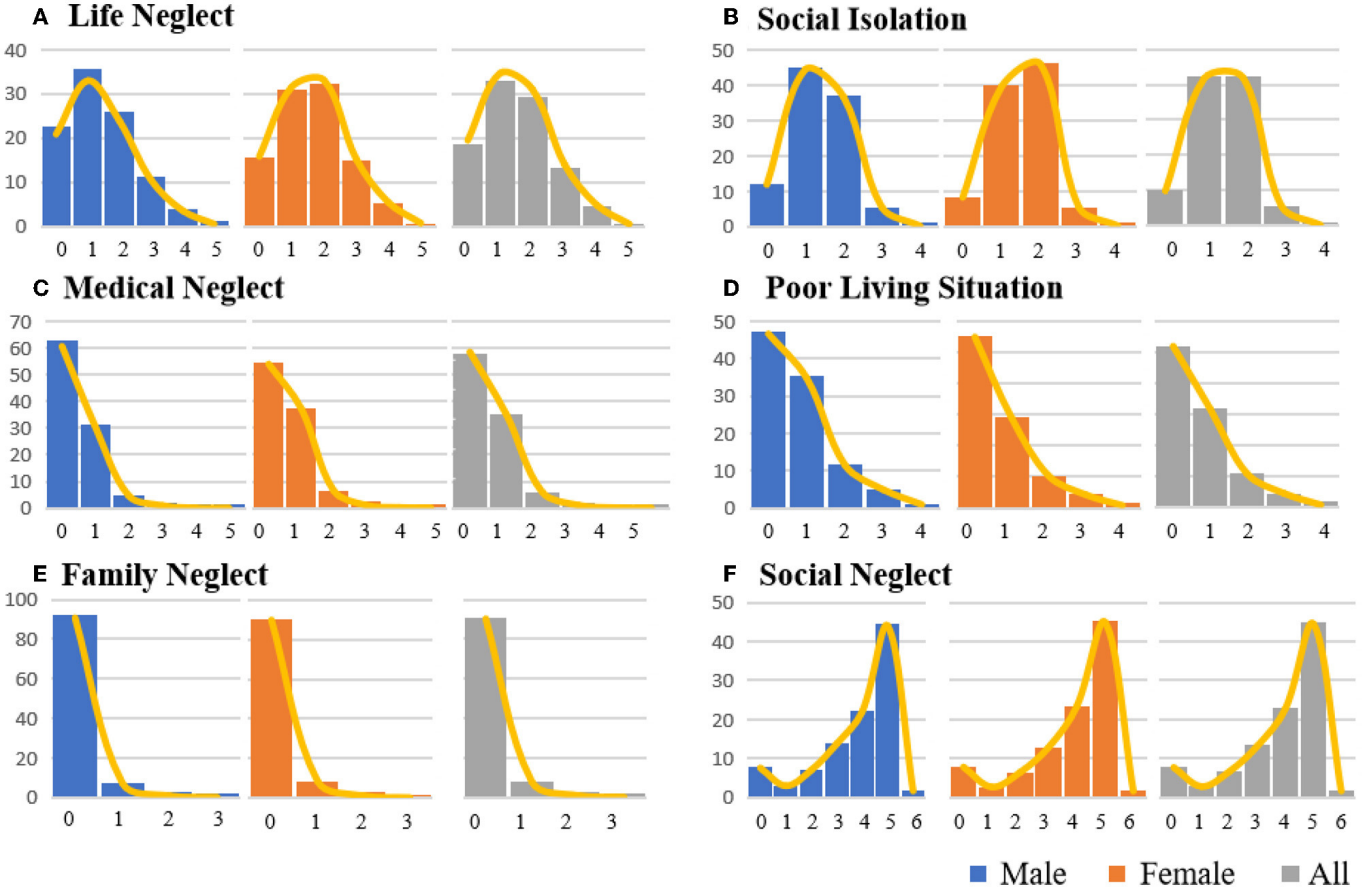
Score distribution by percentage in different domains of neglect. **(A**–**F)** Present the distribution of scores in each domain, and each color represents the same population in CLHLS, blue for male participants, orange for female participants, and gray for the whole population. The line in yellow presents the trend of the distribution in the score of each dimension. The number on the left is the percentage of the population.

The mean (standard deviation) neglect scores, which included three domains of EN (self-neglect, family neglect, and social neglect) were 8.12 (2.85), and the median (quartile 25 and quantile 75) was 8 (6.00 and 10.00). Figure 1 presents the distribution of scores in six dimensions of EN, which showed high consistency among male participants, female participants, and the whole population. It showed that nearly 90% of the participants scored a zero for family neglect, which does not qualify for any of the items. More than half of the participants met <1 of the items for life neglect, medical neglect, and poor living situation. Almost 80% of the population experienced one or two types of social isolation in CLHLS. We found that several characteristics were different among dimensions, including life neglect, social isolation, and medical neglect, which were three dimensions in the self-neglect domain. Participants who were female participants, aged more than 85 years, with a marital status of widow/separated/other, and whose household income was lower than ¥30,000 tend to have higher scores in life neglect and social isolation (Table 2). In addition, the scores for the poor living situation of male participants and those who lived in town or country were both 1 (0,1), which was higher than the score for female participants who live in the city. Furthermore, those who were more than 85 years of age had a higher medical neglect score (0.65 compared with 0.31 in the 60–74 years subgroup and 0.38 in the 75–84 years subgroup, respectively). And found that the average score for poor living situation increased as household income decreased.

**Table 2:** Score in each domain by different subgroup of EN.

	**Number**		**Neglect**	**Self neglect**						
				**Total**	**Life neglect**	**Social isolation**	**Medical neglect**	**Poor living situation**	**Family neglect**	**Social neglect**
**Sex**										
Male	6,912	M (P25, P75)	8.00 (6.00, 10.00)	4.00 (2.00, 5.00)	1.00 (1.00, 2.00)	1.00 (1.00, 2.00)	0.00 (0.00, 1.00)	1.00 (0.00, 1.00)	0.00 (0.00, 0.00)	4.00 (3.00, 5.00)
		Mean	7.87 (2.83)	3.98 (2.1)	1.4 (1.1)	1.36 (0.77)	0.44 (0.64)	0.77 (0.91)	0.09 (0.32)	3.8 (1.54)
Female	8,942	M (P25, P75)	8.00 (7.00, 10.00)	4.00 (3.00, 6.00)	2.00 (1.00, 2.00)	2.00 (1.00, 2.00)	0.00 (0.00, 1.00)	0.00 (0.00, 1.00)	0.00 (0.00, 0.00)	4.00 (3.00, 5.00)
		Mean	8.31 (2.85)	4.37 (2.14)	1.66 (1.12)	1.49 (0.73)	0.55 (0.7)	0.66 (0.89)	0.11 (0.35)	3.83 (1.54)
**Age**										
60–74	3,364	M (P25, P75)	7.00 (5.00, 9.00)	3.00 (2.00, 4.00)	1.00 (0.00, 1.00)	1.00 (1.00, 2.00)	0.00 (0.00, 1.00)	0.00 (0.00, 1.00)	0.00 (0.00, 0.00)	4.00 (3.00, 5.00)
		Mean	6.99 (2.65)	3.13 (1.85)	0.96 (0.88)	1.13 (0.74)	0.31 (0.57)	0.74 (0.90)	0.06 (0.25)	3.80 (1.54)
75–84	6,075	M (P25, P75)	8.00 (6.00, 9.00)	4 (2.00, 5.00)	1.00 (1.00, 2.00)	1.00 (1.00, 2.00)	0.00 (0.00, 1.00)	1.00 (0.00, 1.00)	0 (0.00, 0.00)	4.00 (3.00, 5.00)
		Mean	7.54 (2.74)	3.60 (1.96)	1.21 (1.01)	1.28 (0.74)	0.38 (0.61)	0.74 (0.90)	0.09 (0.31)	3.85 (1.53)
More than 85	6,415	M (P25, P75)	9.00 (7.00, 11.00)	5 (4.00, 6.00)	2.00 (1.00, 3.00)	2.00 (1.00, 2.00)	1.00 (0.00, 1.00)	0 (0.00, 1.00)	0 (0.00, 0.00)	4 (3.00, 5.00)
		Mean	8.88 (2.76)	3.81 (1.54)	1.97 (1.10)	1.65 (0.69)	0.65 (0.71)	0.68 (0.90)	0.12 (0.37)	3.81 (1.54)
**Marital status**										
With spouse	3,364	M (P25, P75)	7.00 (6.00, 9.00)	3.00 (2.00, 5.00)	1.00 (0.00, 2.00)	1.00 (1.00, 2.00)	0.00 (0.00, 1.00)	1 (0.00, 1.00)	0.00 (0.00, 0.00)	4.00 (3.00, 5.00)
		Mean	7.38 (2.65)	3.53 (1.98)	1.18 (1.02)	1.26 (0.75)	0.34 (0.56)	0.75 (0.92)	0.04 (0.22)	3.80 (1.51)
Widowed or divorced	9,453	M (P25, P75)	9 (7.00, 10.00)	5 (3.00, 6.00)	2 (1.00, 2.00)	2 (1.00, 2.00)	1 (0.00, 1.00)	0 (0.00, 1.00)	0 (0.00, 0.00)	4 (3.00, 5.00)
		Mean	8.62 (2.87)	4.65 (2.12)	1.80 (1.12)	1.56 (0.72)	0.62 (0.72)	0.68 (0.89)	0.14 (0.39)	3.83 (1.56)
**Residence**										
City	3,541	M (P25, P75)	8 (6.00, 9.00)	4 (2.00, 5.00)	1 (0.00, 2.00)	2 (1.00, 2.00)	0 (0.00,1.00)	0 (0.00,1.00)	0 (0.00,0.00)	4 (3.00,5.00)
		Mean	7.46 (2.92)	3.78 (2.14)	1.31 (1.12)	1.40 (0.86)	0.58 (0.68)	0.49 (0.74)	0.10 (0.35)	3.58 (1.68)
Town or country	12,313	M (P25, P75)	8 (7.00, 10.00)	4 (3.00, 6.00)	2 (1.00, 2.00)	1 (1.00, 2.00)	0 (0.00, 1.00)	1 (0.00, 1.00)	0 (0.00, 0.00)	4 (3.00, 5.00)
		Mean	8.31 (2.80)	4.32 (2.12)	1.62 (1.11)	1.45 (0.71)	0.48 (0.67)	0.77 (0.93)	0.10 (0.33)	3.89 (1.49)
**Occupation before retirement**										
Manual labor	4,395	M (P25, P75)	8 (6.00, 9.00)	4 (2.00, 5.00)	1 (0.00, 2.00)	1 (1.00, 2.00)	0 (0.00, 1.00)	0 (0.00, 1.00)	0 (0.00, 0.00)	4 (3.00, 5.00)
		Mean	7.58 (2.96)	3.72 (2.12)	1.35 (1.11)	1.34 (0.79)	0.43 (0.62)	0.61 (0.82)	0.09 (0.31)	3.77 (1.65)
Non-manual labor	11,459	M (P25, P75)	8 (7.00, 10.00)	4 (3.00, 6.00)	2 (1.00, 2.00)	1 (1.00, 2.00)	0 (0.00, 1.00)	0 (0.00, 1.00)	0 (0.00, 0.00)	4 (3.00, 5.00)
		Mean	8.32 (2.78)	4.38 (2.11)	1.62 (1.12)	1.48 (0.73)	0.53 (0.69)	0.75 (0.92)	0.10 (0.34)	3.84 (1.50)
**Household income (**¥**/year)**										
0–8,000	4,560	M (P25, P75)	9 (7.00, 11.00)	4 (3.00, 6.00)	2 (1.00, 2.00)	1 (1.00, 2.00)	0 (0.00, 1.00)	1 (0.00, 2.00)	0 (0.00, 0.00)	5 (4.00, 5.00)
		Mean	8.63 (3.04)	4.57 (2.26)	1.66 (1.14)	1.45 (0.73)	0.53 (0.74)	0.93 (1.01)	0.14 (0.40)	3.92 (1.58)
8,000–30,000	3,943	M (P25, P75)	8 (6.00, 10.00)	4 (3.00, 6.00)	2 (1.00, 2.00)	1 (1.00, 2.00)	0 (0.00, 1.00)	0 (0.00, 1.00)	0 (0.00, 0.00)	4 (3.00, 5.00)
		Mean	8.21 (2.74)	4.27 (2.08)	1.62 (1.12)	1.46 (0.7)	0.47 (0.65)	0.72 (0.91)	0.08 (0.31)	3.86 (1.52)
30,000 or more	7,351	M (P25, P75)	8 (6.00, 10.00)	4 (2.00, 5.00)	1 (1.00, 2.00)	1 (1.00, 2.00)	0 (0.00, 1.00)	0 (0.00, 1.00)	0 (0.00, 0.00)	4 (3.00, 5.00)
		Mean	7.75 (2.73)	3.93 (2.04)	1.44 (1.11)	1.42 (0.78)	0.50 (0.64)	0.57 (0.78)	0.08 (0.30)	3.74 (1.52)

Each score presents in both Median (quantiles) and Mean (standard deviation).

### 3.3. Association between different characteristics and domains of neglect

**Figure 2:**
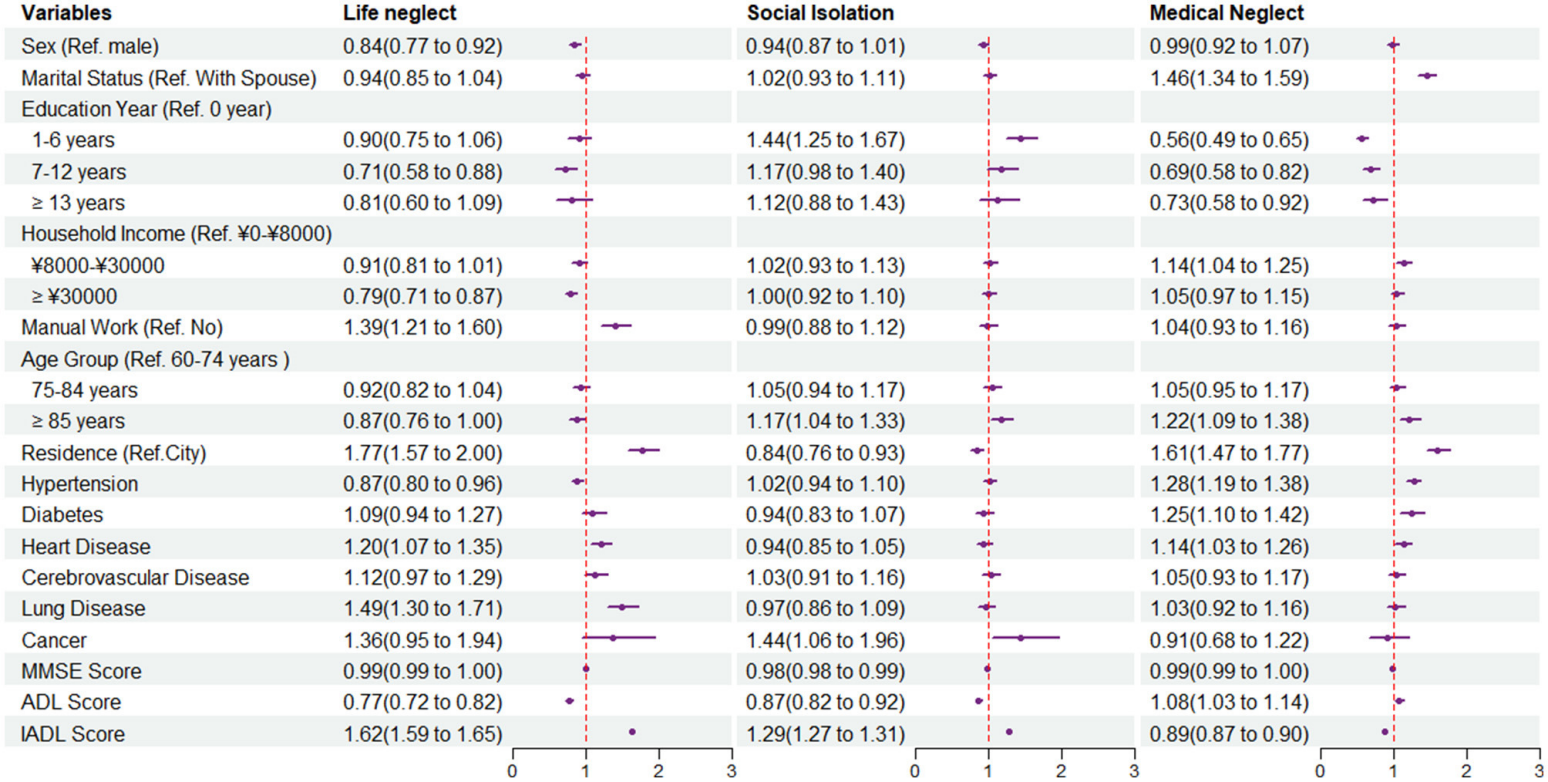
Forest plot of odds ratios (ORs) and 95% confidence intervals of each characteristic in life neglect, social isolation, and medical neglect by multivariate logistic regression.

**Figure 3:**
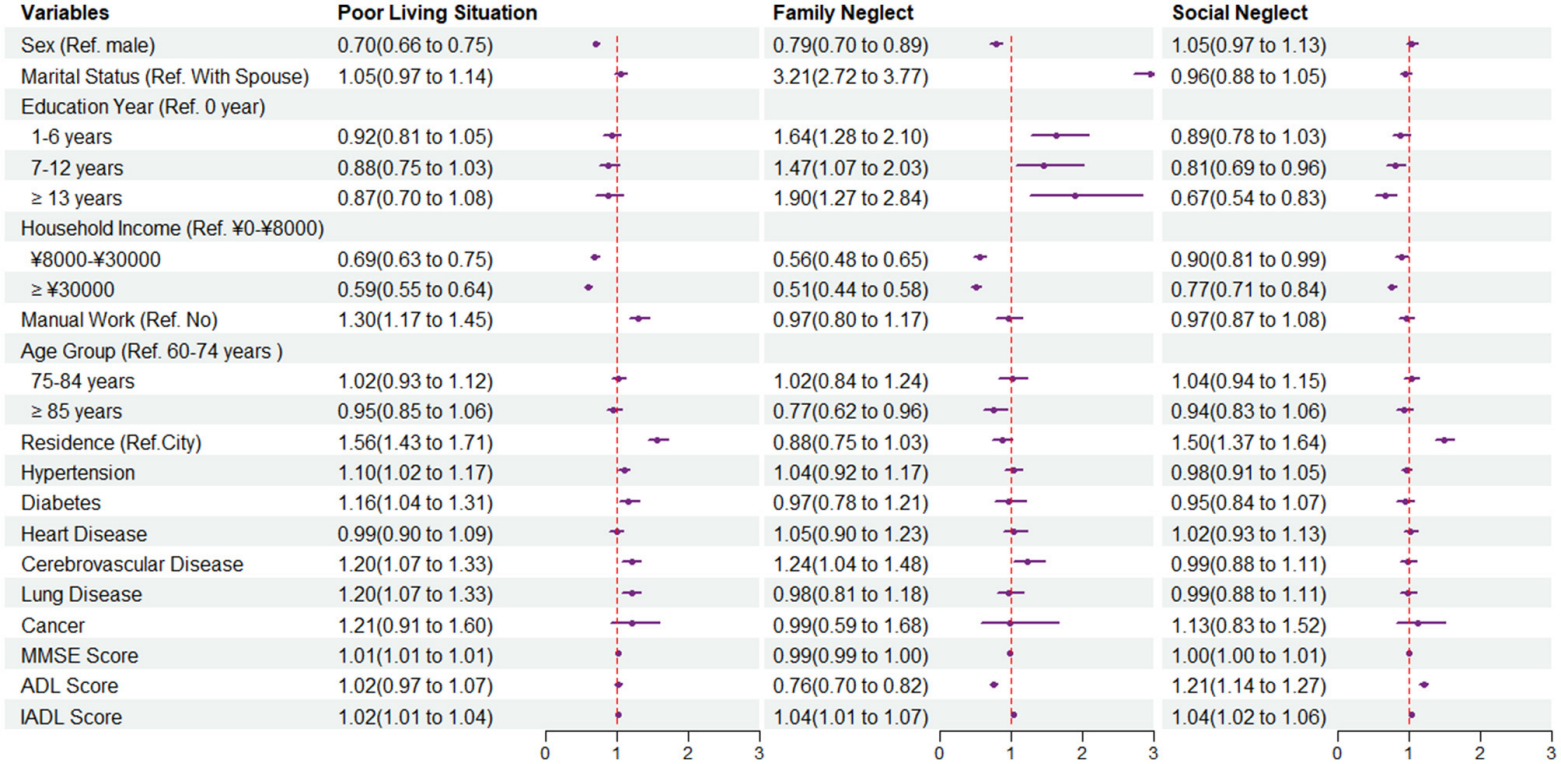
Forest plot of odds ratios (ORs) and 95% confidence intervals of each characteristic in poor living situation, family neglect, and social neglect by multivariate logistic regression.

If the score of a participant was higher than the median score, we regarded the participant as a higher risk group for EN (score 2 in life neglect and social isolation; score 1 in medical neglect, poor living situation, and family neglect; and score 4 for social neglect). We then performed logistic regression to explore the association between different factors and the risk of neglect. Figures 2, 3, and Table 3 presented the OR and 95% confidence interval of each factor. Compared with male participants, female participants were less likely to be neglected in life [OR: 0.85 (0.78, 0.93)], lived in a poor situation [OR: 0.70 (0.66, 0.75)], and had a lower risk to be neglected by family [OR: 0.80 (0.71, 0.90)]. The age-stratified analysis showed a significant difference in medical malpractice among people over 85 years old, with an OR and 95% CI of 1.22 (1.01,1.34). Education year played a significant role in medical neglect and family neglect and showed slightly that participants with higher education degrees were less probably to face a higher risk of social neglect. ADL and IADL scores, which could present the physical function of an individual, might influence neglect in each neglect dimension, except for poor living situations. If participants had a higher score in IADL, which had more difficulties in daily life activities, they would have a higher possibility of being neglected in each dimension. Residence location was also significantly associated with neglect in each dimension and had a different effect in some dimensions. For example, retiring as a manual laborer might be a risk factor in dimensions of life neglect and poor living situations (Figure 3). Living in town and country might have a lower risk of social isolation [OR: 0.84 (0.76, 0.93)] and family neglect [OR: 0.88 (0.75, 1.03)] and had the opposite association with life neglect [OR: 1.77 (1.57, 2.00)], medical neglect [OR: 1.61 (1.47, 1.77)], poor living situation [OR: 1.56 (1.43, 1.71)], and social neglect [OR: 1.50 (1.37, 1.64)]. As for self-reported disease, participants with a chronic disease might suffer more from neglect. Working as a manual worker before retirement could be a high-risk factor for a poor living situation [OR: 1.30 (1.17, 1.45)].

**Table 3:** Odds ratios (OR) and 95% confidence intervals of each characteristic of EN by multivariate logistic regression.

**Category**	**Variables**		**Life neglect**	**Social isolation**	**Medical neglect**	**Poor living situation**	**Family neglect**	**Social neglect**
Demography	Sex	Male	Ref	Ref	Ref	Ref	Ref	Ref
		Female	0.84 (0.77, 0.92)	0.94 (0.87, 1.01)	0.99 (0.92, 1.07)	0.70 (0.66, 0.75)	0.79 (0.70, 0.89)	1.05 (0.97, 1.13)
	Marital status	With spouse	Ref	Ref	Ref	Ref	Ref	Ref
		Without spouse	0.94 (0.85, 1.04)	1.02 (0.93, 1.11)	1.46 (1.34, 1.59)	1.05 (0.97, 1.14)	3.21 (2.72, 3.77)	0.96 (0.88, 1.05)
	Education year	0 year	Ref	Ref	Ref	Ref	Ref	Ref
		1–6 years	0.90 (0.75, 1.06)	1.44 (1.25, 1.67)	0.56 (0.49, 0.65)	0.92 (0.81, 1.05)	1.64 (1.28, 2.10)	0.89 (0.78, 1.03)
		7–12 years	0.71 (0.58, 0.88)	1.17 (0.98, 1.40)	0.69 (0.58, 0.82)	0.88 (0.75, 1.03)	1.47 (1.07, 2.03)	0.81 (0.69, 0.96)
		≥13 years	0.81 (0.60, 1.09)	1.12 (0.88, 1.43)	0.73 (0.58, 0.92)	0.87 (0.70, 1.08)	1.90 (1.27, 2.84)	0.67 (0.54, 0.83)
	Household income	¥0–¥8,000	Ref	Ref	Ref	Ref	Ref	Ref
		¥8,000–¥30,000	0.91 (0.81, 1.01)	1.02 (0.93, 1.13)	1.14 (1.04, 1.25)	0.69 (0.63, 0.75)	0.56 (0.48, 0.65)	0.90 (0.81, 0.99)
		≥¥30,000	0.79 (0.71, 0.87)	1.00 (0.92, 1.10)	1.05 (0.97, 1.15)	0.59 (0.55, 0.64)	0.51 (0.44, 0.58)	0.77 (0.71, 0.84)
	Manual work	No	Ref	Ref	Ref	Ref	Ref	Ref
		Yes	1.39 (1.21, 1.60)	0.99 (0.88, 1.12)	1.04 (0.93,1.16)	1.30 (1.17, 1.45)	0.97 (0.80, 1.17)	0.97 (0.87, 1.08)
	Age	60–74 years	Ref	Ref	Ref	Ref	Ref	Ref
		75–84 years	0.92 (0.82, 1.04)	1.05 (0.94, 1.17)	1.51 (0.94, 1.17)	1.02 (0.93, 1.12)	1.02 (0.84, 1.24)	1.04 (0.94, 1.15)
		≥85 years	0.87 (0.76, 1.00)	1.17 (1.04, 1.33)	1.22 (1.01, 1.34)	0.95 (0.85, 1.06)	0.77 (0.62, 0.96)	0.94 (0.83, 1.06)
	Residence	City	Ref	Ref	Ref	Ref	Ref	Ref
		Town or country	1.77 (1.57, 2.00)	0.84 (0.76, 0.93)	1.61 (1.47, 1.77)	1.56 (1.43, 1.71)	0.88 (0.75, 1.03)	1.50 (1.37, 1.64)
Chronic disease	Hypertension		0.87 (0.80, 0.96)	1.02 (0.94, 1.10)	1.28 (1.19, 1.38)	1.10 (1.02, 1.17)	1.04 (0.92, 1.17)	0.98 (0.91, 1.05)
	Hypertension		1.09 (0.94, 1.27)	0.94 (0.83, 1.07)	1.25 (1.10, 1.42)	1.16 (1.04, 1.31)	0.97 (0.78, 1.21)	0.95 (0.84, 1.07)
	Diabetes		1.20 (1.07, 1.35)	0.94 (0.85, 1.05)	1.14 (1.03, 1.26)	0.99 (0.90, 1.09)	1.05 (0.90, 1.23)	1.02 (0.93, 1.13)
	Heart disease		1.12 (0.97, 1.29)	1.03 (0.91, 1.16)	1.05 (0.93, 1.17)	1.20 (1.07, 1.33)	1.24 (1.04, 1.48)	0.99 (0.88, 1.11)
	Cerebrovascular disease		1.49 (1.30, 1.71)	0.97 (0.86, 1.09)	1.03 (0.92, 1.16)	1.20 (1.07, 1.33)	0.98 (0.81, 1.18)	0.99 (0.88, 1.11)
	Lung disease		1.36 (0.95, 1.94)	1.44 (1.06, 1.96)	0.91 (0.68, 1.22)	1.21 (0.91, 1.60)	0.99 (0.59, 1.68)	1.13 (0.83, 1.52)
	Cancer		0.99 (0.99, 1.00)	0.98 (0.98, 0.99)	0.99 (0.99, 1.00)	1.01 (1.01, 1.01)	0.99 (0.99, 1.00)	1.00 (1.00, 1.01)
Cognitive function	MMSE score		0.77 (0.72, 0.82)	0.87 (0.82, 0.92)	1.08 (1.03, 1.14)	1.02 (0.97, 1.07)	0.76 (0.70, 0.82)	1.21 (1.14, 1.27)
Physical function	ADL score		1.62 (1.59, 1.65)	1.29 (1.27, 1.31)	0.89 (0.87, 0.90)	1.02 (1.01, 1.04)	1.04 (1.01, 1.07)	1.04 (1.02, 1.06)
IADL score		0.87 (0.80, 0.96)	1.02 (0.94, 1.10)	1.28 (1.19, 1.38)	1.10 (1.02, 1.17)	1.04 (0.92, 1.17)	0.98 (0.91, 1.05)

## 4. Discussion

The CLHLS study covers 22 of the 31 provinces in China. Approximately half of the rural and urban counties were randomly selected for this survey. The evaluation of 15,854 older individuals in this study represents a more typical Chinese older adult population. We found that demographic factors such as gender, age, marriage, occupation, income, and residence all have different effects on the six dimensions of EN. In the presence of chronic diseases, reduced cognitive function, and ability of daily activities may adversely affect EN risk.

Based on the research conducted domestically and abroad, our investigation encompassed 28 items of EN and six dimensions of neglect, namely, life neglect, social isolation, medical neglect, poor living situation, family neglect, and social neglect. The first four dimensions represent the areas of self-neglect, bonding family neglect, and social neglect together as the three domains of EN. A higher total score corresponded to a higher EN level. The median of self-reported EN in older adults ranged from 6 to 10, with an overall median of 8. A similar result was observed in a cross-sectional study conducted in China in 2020. Chen et al. used the Revised Conflict Tactics Scale (CTS2), which contains 20 items, to assess EN. The mean EN was 8.26 ± 11.9 (11). In addition, in a cross-sectional study of EN in a U.S. community-dwelling population (the Chicago Health and Aging Project [CHAP]), 15 items were used to evaluate EN, with a maximum cumulative score of 45 points, and a mean EN score of 15.9 (SD = 10.4, range 0–45) (*N* = 1,094) (21). In another community study from Europe, EN was evaluated based on 16 terms from the Self-Reported Neglect Scale (SRNS); the median total EN was 12.3, with a range of 8.4–16.9 (*N* = 2,443) (22). However, an EN screening scale has only been developed in recent years, and uniform standards are still lacking. Furthermore, our results suggest that older adults over the age of 85 are more likely to be subject to medical neglect. The majority of our study population is the elderly population of villages and towns. There is still a problem of under-allocating medical resources in these areas. It is believed that the situation will gradually improve with the support of government policies and the construction of medical resources. In addition, the elderly are generally disabled, bedridden, cognitively impaired, suffer from basic diseases, and other health problems and require special care and attention. As they grow older and the burden on their families increases, the medical needs of the elderly may not be attended to in a timely manner. There is a need to strengthen the promotion and education of social filial piety and respect for the elderly, increase community services for the elderly, provide counseling and help for the families of the elderly, and reduce the occurrence of medical neglect.

In addition, we discussed the distributional characteristics of EN subtypes in the elderly population of Chinese communities. Our results found large differences in the distribution of scores for family neglect, life neglect, medical neglect, and social isolation. The evaluation content of these dimensions is independent of each other and thus may affect the distribution. In addition, we believe that the differences in the distribution of these dimensions also reflect the differences in the cultural habits of Chinese society toward the elderly. For example, regarding the low number of elderly people reporting family neglect, we conjecture that this may occur for some reasons: Compared with Western countries, sociocultural norms related to filial piety, home-based care for older adults, and respecting one's elders are deeply ingrained in Chinese families (23). Many older victims of EN feel ashamed and unwilling to report family neglect by their own children to the public, thus conducting investigations in the community is difficult (24). Finally, the distribution and size of the studied population also affect the distribution of EN dimensions. In future studies, we will take a closer look at the social reasons for these differences.

In our study, the average age of the older adults was 85 years, and the majority were older women. In addition, older women accounted for a large proportion of the living without spouse group (72.9%). We found that women are less vulnerable to neglect in life, in their residential environments, and in families, and we believe that this is related to the role of women in the family. For those who lived without a spouse, medical neglect and family neglect are more likely to happen. Older adults are more vulnerable and require intimate support from their households. Separation from a spouse imposes enormous psychological stress on older adults, sometimes leading them to overlook their symptoms and medication. Changes in the family environment affect the efficiency of medical management (25). We found that hyper-elderly and elderly individuals who are unskilled at using assistive tools lead to a narrowing of social network size, resulting in a paucity of social interactions. Social isolation gives rise to a sense of loneliness, leading to self-neglect as a behavioral reaction to psychological distress (15). Most older adults come from towns or the countryside; engage in manual work; and remain illiterate or receive a basic level of education. These characteristics are partly historical and are in line with social development. Older adults living in towns and counties are more vulnerable to life neglect, medical neglect, poor living conditions, and social neglect. Zhao et al. found that poor quality of life was significantly associated with self-neglect in rural older adults in the Anhui Province of China (23). All these reflect the living environment and culture of towns and counties, and the medical conditions and social service conditions require further attention and improvement. Manual workers are prone to the risk of EN of life and poor living conditions. There are several possible reasons for this. First, heavy physical work or manual labor can give rise to physical and cognitive impairment, worsening chronic illness, and long-term disability, which may increase the risk of self-neglect (24). Second, manual workers often have more family responsibilities, psychological stress, long working hours, and strained family relationships. These factors may lead to poor lifestyle habits, ignorance of their own health, and a lack of family support (26). Good education level and household income have a positive effect on avoiding EN. We found that older adults living in towns and counties select more options for medical and social neglect. The uneven development of medical and social assistance for urban and rural older adults remains a challenge. Community services in big cities have matured rapidly, creating home services, healthcare, and recreational activities and generating patterns of community engagement. In comparison, the development of community services in rural towns is slow and remains challenging (27). This finding highlights the urgent need for public social services for older adults in more rural locations. Future longitudinal clinical studies of older adults with each dimension of EN are needed.

In addition, we evaluated the basic ADL and IADL. ADL refers to the self-care skills necessary for basic living, including feeding, grooming, dressing, toileting, transferring, and walking. IADL refers to advanced skills needed to live independently in the community, including transportation, shopping, housekeeping, and medical administration. We found that the decline in the ability to perform daily tasks is an important factor contributing to all dimensions of EN. Impairment in any measure of daily activity capacity is of significant clinical relevance and may indicate difficulties in daily self-care and reliance on others for basic assistance (28). Our findings are consistent with those of previous studies. Howe et al. investigated 2,340 participants from the NSHAP project (2015–2016) and found that older adults with ADL/IADL limitations tended to have worse family support and a higher risk of EN (29). Dong et al. examined 5,570 participants in the CHAP project (1993–2010) and found that a decline in ADL/IADL was associated with an increased risk of self-neglect (OR 1.05, 95% [CI] 1.03–1.07, *p* < 0.001) and greater EN severity in a community-dwelling population (15). Kong et al. analyzed 9,691 community-dwelling older Koreans from the ECWN project (2009) and found that ADL/IADL limitations were associated with greater assistance requirements from family members and were also important factors affecting family neglect (30). These results indicate that improving daily living ability and mitigating dependence on daily assistance have positive implications for community-dwelling older adults (31). Further large-scale clinical studies are warranted to confirm our results.

Finally, we analyzed chronic diseases common in old age, including hypertension, diabetes, heart disease, lung disease, cerebrovascular disease, and cancer. We found that neglect of life and living environment was common among older adults with hypertension, heart disease, diabetes, and lung disease. The probable reason we conjecture is that the onset of these diseases is intimately connected with the habits and circumstances of life. In addition, older people suffering from certain chronic diseases are prone to medical neglect, which may be related to the large rural population in our study and the current social situation of unbalanced development of medical conditions in urban and rural areas, as previously mentioned. Elderly patients with cerebrovascular diseases, such as stroke, are more likely to receive family neglect, which may be associated with hemiplegia, disability, and difficulties in care due to cerebrovascular disease. Cognitive function is an important factor in the self-management and self-care abilities of older adults. We found that older adults with better cognitive function were less likely to experience neglect. Chronic disease and cognitive decline are thought to be strongly associated with elder maltreatment, and our findings are consistent with previous studies (18, 32).

The health problems of the older victims of EN have become prominent. Regrettably, there is insufficient evidence to recommend EN screening in the community. In many instances, medical institutions and rehabilitation and nursing facilities ignore this problem and do not take appropriate action to prepare older adults, because of insufficient clinical information. How can EN risk be detected in the community? Our study provides a unique window into this issue, helping us to better understand the different factors associated with various types of EN. As a professional with the opportunity for the early detection of abuse, clinicians must be prepared to recognize the signs and concealed features of EN and try to create effective intervention plans with other social security groups. In such cases, the physician's role is to provide strong evidence from a health survey that supports the presence of EN. Due to the complex nature of EN cases, physicians can also serve as a catalyst for the social assistance of older adults in the local community.

Our study had some limitations. First, EN events might be under-reported, owing to their implicit and hidden features. Over time, an increasing number of EN cases in the CLHLS study are expected to be detected and collected. Second, although EN studies have been emphasized in recent years, owing to historical reasons, there were no mature EN self-assessment tools available when our project was initiated. Moreover, most existing EN assessment tools are based on other self-report questionnaires, and we hope that more self-report questionnaires with high reliability and validity that are suitable for Chinese older adults will be developed in future. This study lays the foundation for future research. Third, the CLHLS study was a nationwide cross-sectional study. Further analyses are needed to explore the relationships between the dynamic evolution of social–economical–cultural contexts and EN. Fourth, our study did not adequately cover all factors that influence EN in the available studies. Therefore, this study did not directly provide a precise measure or specific indicators of EN. It remains difficult to objectively compare EN differences between different populations in cross-cultural environments. Fifth, the clinical relevance of EN severity remains largely unknown. The health side effects of increasing EN severity warrant further validation. Despite these limitations, our research is the largest community study of older adults to comprehensively evaluate EN in China. In this study, we surveyed residents of elderly communities in China from several dimensions of EN. These results not only provide a further foundation in understanding the conceptual framework of EN applicable to older adults in China but also serve as a basis for the development of targeted EN assessment criteria and prevention strategies in future.

## Data availability statement

The datasets presented in this study can be found in online repositories. The names of the repository/repositories and accession number(s) can be found below: http://opendata.pku.edu.cn.

## Ethics statement

The studies involving human participants were reviewed and approved by the 2018 wave of CLHLS study was approved by the Biomedical Ethics Committee of Peking University (IRB00001052-13074). The patients/participants provided their written informed consent to participate in this study.

## Author contributions

All authors listed have made a substantial, direct, and intellectual contribution to the work and approved it for publication.
