# Supplementary material for: Prevalence and correlates of elder neglect in the community-dwelling Chinese population: New evidence from the CLHLS study
Source: Front Public Health. 2023 Mar 13;11:1123835. doi: 10.3389/fpubh.2023.1123835 (PMC10040648; doi:10.3389/fpubh.2023.1123835)
Supplement: Supplementary file 1 [file Table_1.pdf]

Supplementary table 1
Items of elder neglect in the CLHLS study.

Dimensions of EN Assessment	Assessment Questions	Answers to questions
**Self neglect**		
**Life neglect**		
Unwilling to cook	Cook alone (E9)	Can (Answer 1)
		Can not (Answer 2-3)
Avoid fresh fruit and vegetables	Eat fresh vegetables and fruits on a regular basis(D3-1,2)	Yes (Answer 1-2)
		No (Answer 3-4)
Irregular exercise	Take regular exercise (D9-1)	Yes
		No
Poor hygiene	Able to wash, bathe or dress (E1,2,10)	Yes (Answer 1)
		No (Answer 2-3)
**Social isolation**		
Lack social interaction to others	Visit and socialize with friends (D11-2c)	Yes (Answer 1-4)
		No（Answer 5）
unwillingness to participate in social activities	Participate in social activities(D11-8)	Yes (Answer 1-4)
		No（Answer 5）
Unwilling to share own information	Be willing to confide in people(F11-2)	Yes (Answer 1-9)
		No（Answer 10）
Uncooperative	Distrust of those around(*B2-7)	Yes (Answer 1-3)
		No（Answer 4-5）
Solve daily problems on one own	The community provides residential care (F14-1)	Yes
		No
**Medical neglect**		
Untimely access to emergency care	Get prompt treatment when get sick(F6-1)	Can
		Can not
Unaccompanied clinic visits	Unaccompanied to the hospital(F6-1.0)	Yes (Answer 4-5)
		No（Answer 1-3）
Unattended care for illness	Sick and unattended at the hospital(F5)	Yes (Answer 12)
		No（Answer 1-11）
Irregular physical examination	Annual physical examination (F6-5.2-b)	Yes
		No
**Poor living situation**		
Poor kitchen ventilation	The kitchen is ventilated while cooking(A5-3.7)	Yes (Answer 2-4)
		No（Answer 1）
Musty taste in the house	The room smells musty (A5-3.5)	Yes (Answer 1)
		No（Answer 2）
Leaky roof	Leaks and flooding in home(A5-3.4)	Yes
		No
Untidy home environment	Tidy environment(*B2-2)	Yes (Answer 1-2)
		No（Answer3-5）

**Family neglect**		
Can't get along with spouse	Can't get along with spouse (F4-3)	Yes (Answer 1-2)<br>No（Answer 3）
Live alone due to no assistance from children	The child is unable to care for the parent(A5-8)	Yes (Answer 1)<br>No（Answer 2）
Unattended and live in nursing homes	Living in a care home because no children to care for (A5-6.0)	Yes (Answer 1)<br>No（Answer 2-4）
Reluctant caregivers	Caregivers are reluctant to take care of daily life(E6-2)	Yes (Answer 2,4)<br>No（Answer 1,3）
Unmet ADL needs	The daily care of the caregiver cannot meet the needs (E6-5)	Yes (Answer 3)<br>No（Answer 1-2）
**Social neglect**		
Relatives unable to resolve daily problems	Can find someone to solve daily problems(F11-3)	Yes (Answer 1-9)<br>No（Answer 10）
Cannot provide social care	Community offers door-to-door service(F14-3)	Yes<br>No
Doctors house calls and medicine delivery	Home medical service(F14-2)	Yes<br>No
Shopping help	Shopping help (F 14-4)	Yes<br>No
Legal aid	Legal aid (F14-6)	Yes<br>No
Handling family disputes	Handling family disputes(F14-8)	Yes<br>No
